# Molecular Dynamics Simulation of Low-Cycle Fatigue Behavior of Single/Polycrystalline Iron

**DOI:** 10.3390/nano15030217

**Published:** 2025-01-29

**Authors:** Tianyu Zhang, Jinjie Zhou, Jinchuan Shen

**Affiliations:** 1 School of Mechanical Engineering, North University of China, Taiyuan 030051, China; m15235053235@163.com (T.Z.); shenjinchuanchina@163.com (J.S.); 2 Shanxi Key Laboratory of Intelligent Equipment Technology in Harsh Environment, North University of China, Taiyuan 030051, China

**Keywords:** single-crystal iron, polycrystalline iron, cyclic loading, dislocation evolution, molecular dynamics

## Abstract

The fatigue plastic mechanism and dislocation characteristics of engineering materials are the key to studying fatigue damage. In this study, the molecular dynamics (MD) method was employed to investigate the microstructural characteristics and fatigue mechanical properties of both single-crystalline and polycrystalline iron under varying strain amplitudes associated with cyclic hardening, cyclic softening, and cyclic saturation. The occurrence, accumulation, and formation process of the local plastic fatigue damage of monocrystalline/polycrystalline iron under fatigue load are discussed. The local plastic initiation and accumulation of single-crystal iron occur at the intersection of slip planes, which is the dislocation source. The 1/2<111> dislocation plays an important role in the fatigue plastic accumulation of single-crystal iron. Polycrystalline iron undergoes grain rotation and coalescence during cyclic loading. The grain size responsible for plastic deformation gradually increases. The initiation and accumulation of local plasticity occurs at the grain boundary, which eventually leads to fatigue damage at the grain boundary.

## 1. Introduction

Body-centered cubic (BCC) metals, including iron (Fe), molybdenum (Mo), tungsten (W), and niobium (Nb), are extensively utilized in various aspects of daily life and industrial manufacturing due to their remarkable plasticity and ductility across a broad spectrum of deformation conditions and temperatures [1,2,3]. Owing to the plentiful availability of iron ore and well-established smelting techniques, body-centered cubic iron and its alloys have emerged as some of the most crucial metal structural materials. However, the industrial application of body-centered cubic iron is significantly constrained by its relatively low strength and hardness. Therefore, improving the comprehensive mechanical properties of BCC-Fe and its alloys has important scientific and technical significance.

Fatigue fracture refers to the phenomenon of defect propagation and damage accumulation resulting from repeated stress. This process influences the lifespan of mechanical components exposed to cyclic loads, thereby significantly impacting the reliability of mechanical systems and production costs. During the service life, the components subjected to relatively high stress must withstand numerous cycles. Fatigue fracture can be classified into low-cycle fatigue, high-cycle fatigue and very high-cycle fatigue based on the number of loading and unloading cycles that a component can undergo during its expected service life. Plastic deformation induced by fatigue accumulation is the key to evaluating fatigue damage. In order to reveal the microstructure evolution and plastic deformation in the process of metal fatigue crack propagation, experiments and theories were carried out to study them [4,5,6,7,8]. Hu et al. [9] investigated the influence of loading frequency and mode on the fatigue strength and fatigue life of a certain high-strength steel through thermal treatment methods such as rotary bending (52.5 Hz), electromagnetic resonance (120 Hz), and ultrasonic (20 kHz). The results show that the loading frequency effect is the result of increasing strain rate and temperature. In order to find the correlation between macroscopic experiments and nanoscale atomic results, Michal et al. [10] presented new experimental results for the fracture test of BCC ferrosilicon single crystals with edge cracks under cyclic loading at room temperature. The experimental results are in good agreement with the basic prediction of fatigue crack growth by the continuum model and molecular dynamics (MD) simulation. Takayuki [11] studied the effect of cyclic softening performance on fatigue crack growth behavior. Ferritic and ferritic pearlite steels with different cyclic softening properties were prepared by the cold rolling process. Fatigue crack growth was tested with CT samples, and the fatigue crack growth rate decreased with the increase in cold reduction rate. The results show that the suppression of fatigue crack growth by cold rolling is mainly due to the suppression of crack opening by cyclic softening near the crack tip. Paul [12] conducted a low-cycle fatigue test of 304LN stainless steel in ambient air at room temperature. The single-axis ratchet behavior of the material was studied under engineering and true stress control modes. The results show that the cyclic hardening/softening behavior of the material under low-cycle fatigue and ratchet is not only related to the material, but also to the loading conditions. The researchers also looked at the macroscale. Linear elastic fracture mechanics and the finite element method were used to analyze and numerically identify fatigue crack growth on a macroscopic scale and were recommended for practical applications [13,14]. Podrug [15] established a computational model to determine the influence of moving loads on the fatigue process including the crack initiation and crack growth periods. The crack initiation direction was predicted by the critical plane method. The numerical results are close to the experimental results. However, even though stress–strain is elastic on the macro scale, the material may also suffer plastic deformation on the microscale.

Unlike continuum mechanics, which includes linear elastic fracture mechanics and elastoplastic fracture mechanics, molecular dynamics (MD) simulations treat materials as many discrete, discontinuous, interacting atoms and molecules. Therefore, the properties of the material can be better revealed from the perspective of the microscopic scale. In recent years, in the field of materials science, researchers have applied MD simulation to study the low-cycle fatigue mechanical behavior of FCC and BCC metals. Veerababu [16] et al. conducted a molecular dynamics simulation of the low-cycle fatigue behavior of nanowire copper at 10 K. The results show that the cyclic stress response of the model is closely related to the microstructure, and the cyclic characteristics of the model are determined by the competition between HCP atoms and disordered atoms. Wu [17] et al. established titanium models with different twin boundary spacing and used molecular dynamic methods to study the fatigue mechanical behavior of the models under different loads and twin spacing. The results show that the deformation mechanism of the twin titanium model is different under tensile load and compression load. The interaction between the twin and the dislocation is dominant under tensile load, and the dislocation slip is dominant under compression load. Luo et al. [18] studied the effect of grain size on the strain-controlled low-cycle fatigue mechanical properties of a CoCrFeMnNi high-entropy alloy. Through a molecular dynamics simulation, it is observed that the slip from the dislocation in place to the twin causes a different cyclic behavior of the model. The slip from the plane dislocation to the wavy dislocation dominates the deformation behavior. Zhou [19] et al. studied the tensile and low-cycle fatigue behavior of Ti nanowires at 300 K by the molecular dynamics method. The results show that the cyclic characteristics of the model are determined by the competition mechanism of different types of atoms in the fatigue process, and the tensile yield strength of the model changes with the crystal direction. Yasbolaghi [20] established single-crystal and polycrystal models using molecular dynamics methods and simulated the low-cycle fatigue crack behavior of the two models under cyclic loading/unloading conditions. The results show that crack initiation occurs during cyclic loading near the tip, and fatigue crack propagation occurs at the last stage of cyclic recording. The microstructure, such as dislocation, can reduce the crack propagation rate and prevent the crack. Sainath et al. [21,22] pointed out that the low-cycle fatigue deformation behavior of BCC-Fe single crystals depends on the size and orientation of the single crystal. Both experimental and theoretical studies show that crystal orientation has a considerable influence on the deformation mechanism of BCC crystals, in which the competition and cooperation of different deformation modes determine the mechanical behavior related to orientation. Sevillano [23] obtained the volume change of α-Fe nanocrystalline samples with <1-10> random fiber texture under low-cycle fatigue plastic deformation at 300 K by a molecular dynamics (MD) simulation. The results show that the superposition of the contribution of the structural change to the mass density change caused by a different plasticity can reasonably explain the observed complex behavior. The main factors are grain size, grain boundary structure, dislocation density, and point defect density.

Generally speaking, the process of plastic deformation can be uniformly described as the kinetic behavior of atoms [24,25]. Furthermore, during the plastic deformation of alloys, similar to their strength and ductility, their thermodynamic and kinetic behaviors are mutually exclusive [26]. The dynamics of dislocation behavior dominates the plastic deformation mechanism of BCC-Fe, thus determining the mechanical properties of BCC-Fe [27]. In this paper, the low-cycle fatigue behavior and microscopic deformation mechanism of single-/polycrystalline iron under cyclic loading were studied by the molecular dynamics method. The low-cycle fatigue behavior and deformation mechanism under different strain amplitudes are discussed. Most studies are limited to considering constant loads with stress or strain amplitudes. In this study, a three-dimensional cylindrical atomic model of single-/polycrystalline iron was established. And the effects of different strain amplitudes on the fatigue mechanical behavior of single-/polycrystalline iron were analyzed. The low-cycle fatigue deformation mechanism of single-/polycrystalline iron under different strains is discussed from the atomic point of view. Firstly, the yield stress of single-crystal/polycrystalline iron under tensile and compressive conditions was obtained by a molecular dynamics (MD) simulation. Three different strain amplitudes of single-/polycrystalline iron were determined by the yield stress. Finally, the dislocation motion of single-/polycrystalline iron with different strain amplitudes is analyzed from several angles. There is little open field literature on the deformation mechanism of single-/polycrystalline iron at the atomic length scale. Our study elucidates the plastic deformation behavior of single-/polycrystalline iron at the atomic scale under cyclic loading conditions and studies the cyclic plastic deformation under defects such as dislocation density, complete spiral dislocation, rootless edge dislocation, and deformation twins during plastic deformation. It is believed that this discovery will greatly contribute to the understanding of the characteristics of cyclic plastic deformation of mono/polycrystalline iron materials.

## 2. Materials and Methods

**Figure 1:**
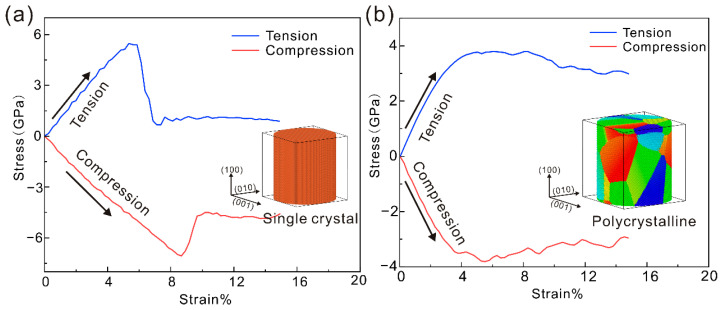
Tensile and compressive mechanical responses of (**a**) monocrystalline iron and (**b**) polycrystalline iron at a loading rate of 5 × 10^9^ (Blue is the tensile curve, red is the compression curve).

All the molecular dynamics simulations in this paper were conducted using LAMMPS (2 Aug 2023) [28]. The EAM potential function was employed to describe the interatomic interactions in both single-crystal and polycrystalline iron [29]. For the establishment of a polycrystalline iron model, Atomsk was used to establish a square polycrystalline iron model of 200 Å × 200 Å × 200 Å, and then the LAMMPS cylinder command was used to modify the model to obtain a columnar iron model. The initial model contained 12 grains. In the present study, the initial cylindrical iron model has a radius of 100 Å and a height of 210 Å along the z-direction. The box contains about 560,000 atoms, and the two models are shown in Figure 1. The coordinate system of the model is x (100), y (010), z (001). All MD simulations were performed at 300 K to model the fatigue characteristics of both single-crystalline and multicrystalline iron under ambient temperature conditions. The conjugate gradient method was employed to achieve energy minimization of the initial system. Subsequently, the NVT ensemble was utilized for a 100 ps relaxation period to ensure that the system’s energy reached a stable state. Due to the limitation of the atomic position, the model does not form microscopic defects when subjected to stresses and strains less than the yield point, even after tens of thousands of reciprocating loads. Therefore, aperiodic and contractile boundary conditions are used in all three directions. The relevant details of the fatigue test are as follows: it first sets the basic conditions of the simulation through a series of commands, including the unit system, boundary conditions, atomic styles, and so on. Then, instead of creating the atomic model from scratch, it initializes the mock system by reading the restart file a.100,000.restart. During the simulation, the program defines multiple regions and groups of atoms for subsequent loading and operations. The region command defines the upper and lower boundary regions and mobile regions. The group command creates atomic groups based on these regions, such as down, top, boundary, and mobile. These atomic groups play a key role in the subsequent loading process. The core of the fatigue test is the cyclic loading process. The program uses a variable, loop 50, to set up 50 loops, and each loop includes two phases of compression and stretching. At each stage, the fix command is used to load a linear shift at a rate of ${erate}, calculated from the z-direction change in the length of the simulated box. It then runs 20,000 steps per load by running the run 20,000 command. To accurately record the data during the simulation, the program calculates two strains: strain1, based on changes in the z-direction length of the simulated box, and strain2, related to time steps and strain rates. At the same time, the atomic stress is calculated by the compute command, and the mean value of the stress component is calculated by the variable command. After average processing by the fix command, these stress–strain data are output to stress_strain1.txt and stress_strain2.txt files, which provide data support for the subsequent analysis of the fatigue properties of materials. In addition, the program also records the trajectory information of atoms through the dump command in order to observe the movement of atoms during the cyclic loading process. During loading, the moving atomic group is temperature-controlled by the fix NVT command at 300 K, simulating fatigue test conditions at a constant temperature. In terms of the initial condition setting, the program sets the initial speed of the upper and lower boundary atoms to 0 through the velocity command and sets the force of these atoms to 0 through the fix command, ensuring that the initial state of the loading process is stable. These are the details of performing fatigue loading in an MD simulation.

For visualizing the simulation outcomes, the Open Visualization Tool (OVITO) [30] was employed. When depicting the deformation process, the equivalent stress was computed in accordance with the von Mises yield criterion. The von Mises stress is calculated through the following equation [31,32,33]:

(1) $$\sigma_{e}=\left(1/\sqrt{2}\right){\left[{\left(\sigma_{x}-\sigma_{y}\right)}^{2}+{\left(\sigma_{y}-\sigma_{z}\right)}^{2}+{\left(\sigma_{z}-\sigma_{x}\right)}^{2}+6\left({\tau_{xy}}^{2}+{\tau_{xz}}^{2}+{\tau_{yz}}^{2}\right)\right]}^{1/2}$$

where $\sigma_{x}$, $\sigma_{y}$ and $\sigma_{z}$, respectively, denote the average equivalent stress components in the *x*, *y* and *z* directions, and $\tau_{xy}$, $\tau_{xz}$, $\tau_{yz}$, respectively, denote the three shear stress components in the *xy*, *xz*, *yz* in the plane of the material. For the purpose of identifying the high-stress regions, the atomic strain approach [34] was employed to color the atoms. To characterize the microstructural evolution of the single-crystal and polycrystalline iron under fatigue loading, the central symmetry parameter (CSP) was employed as an analytical tool [35]:

(2) $$CSP=\left(1/{D_{0}}^{2}\right)\sum_{j=1,6}{\left|R_{j}+R_{j+6}\right|}^{2}$$

where $R_{j}$ and $R_{j+6}$ denote the six nearest reciprocal lattice vectors of the pair bonds in the system, and $D_{0}$ represents the distance between two adjacent atoms. Furthermore, for the purpose of identifying the evolution of dislocations in the sample, the dislocation extraction algorithm (DXA) proposed by Stokowski was employed.

As depicted in Figure 1, a triangular waveform loading mode with a strain ratio of R = −1 (wherein R represents the ratio of the minimum strain to the maximum strain in each cycle) was utilized in this research to perform fatigue loading on single-crystal iron and polycrystalline iron models. Four layers of atoms are, respectively, fixed at the upper and lower ends of the model, and the deformation of the model is controlled by setting a constant movement velocity for the fixed atomic layer at the upper end. The commonly employed loading rate range lies between 1 × 10^7^ and 1 × 10^10^ in molecular dynamics simulations. Previous studies have verified that the enhancement of the strain rate does not change the deformation mechanism of materials [36]. Shen et al. [37] compared the stress–strain curves of monocrystalline aluminum and polycrystalline aluminum under different strain rates during uniaxial tensile, verifying the feasibility of the loading rate of 5 × 10^9^ s^−1^ by verifying the mechanical response and deformation mechanism under the two loading rates. T.-N. Vu et al. [38] established a high-entropy alloy model and discussed the effects of four loading rates of 1 × 10^8^, 5 × 10^8^, 1 × 10^9^, 5 × 10^9^ and 1 × 10^10^ on the mechanical properties of the high-entropy alloy. The results showed that the strain rate changed the evolution of the original substructure during the deformation process. But the differencein atomic structure evolution induced by different strain rates is not significant. The analysis by Fan et al. [39] also shows that increasing the strain rate can promote more atoms to participate in deformation, making the deformation of the system more uniform, and thus improving the yield strength. All these prove that the strain rate is feasible in simulation. At the same time, the higher loading rate means that the model can experience greater strain changes in a shorter time, and the shortened response time makes the model reach the steady state or saturation state faster during the cyclic loading process. Therefore, the fatigue loading rate of 5 × 10^9^ is adopted in this paper, and the strain amplitude range of the fatigue load is determined.

As can be observed from Figure 1, the tensile yield strain and compressive yield strain of the single-crystal iron are 5.3% and 8.6%, respectively, and those of polycrystalline iron are 5.1% and 3.8%, respectively. The stress–strain curve of the single-/polycrystalline iron is observed by increasing the strain on the basis of the yield strain of the single-/polycrystalline iron under tension and compression until the single-/polycrystalline iron begins to undergo plastic deformation. For the single-crystal iron, the chosen strain amplitudes were Δε = 6%, 6.5%, 7%, whereas for the polycrystalline iron, they were Δε = 5%, 5.5%, 6%. Under each strain amplitude, 50 cycle tests were carried out.

## 3. Results

### 3.1. Fatigue Mechanical Response of Single-Crystal Iron

**Figure 2:**
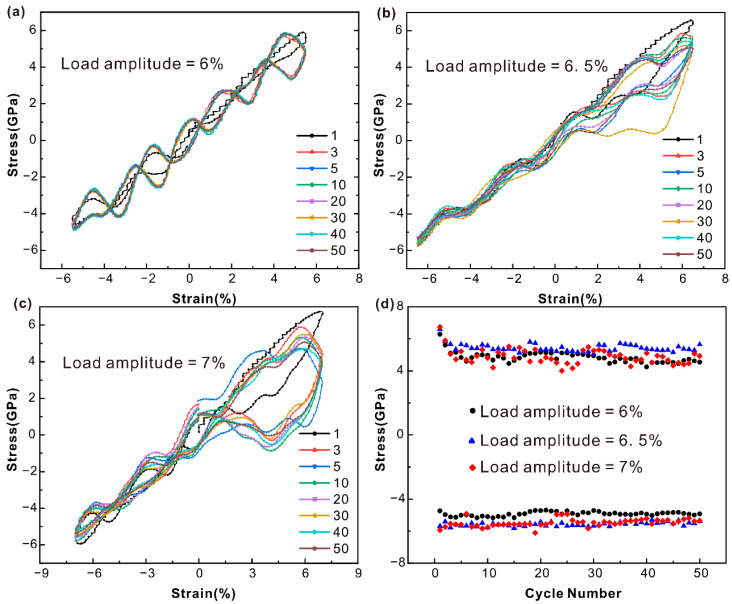
The cyclic stress–strain response curves of single-crystal iron under diverse strain amplitude conditions of (**a**) 6%, (**b**) 6.5%, (**c**) 7%, and (**d**) the variation relationship of tensile and compressive peak stress with the number of cycles.

Figure 2 shows the cyclic stress–strain curves of the single-crystal iron model at the 6%, 6.5%, and 7% strain amplitudes. As can be seen from Figure 2a, the single-crystal iron enters a relatively stable state after cyclic softening when the strain amplitude is 6% and the number of cycles increases. The shape of the hysteresis curve remains basically unchanged and the stress reaches a saturation value. The black dots in Figure 2d correspond to the peak tensile and compressive stresses of the 6% strain amplitude. The monocrystalline iron underwent tensile cycle softening and reached cyclic saturation after 15 cycles. The compressive cyclic stress is stable at a certain level. The hysteresis curve of the single-crystal iron at a strain amplitude of 6.5% shows obvious cyclic hysteresis, as shown in Figure 2b. The tensile extreme stress of monocrystalline iron shows a tendency of cyclic softening at a strain amplitude of 6.5%, as shown in the blue triangle in Figure 2d. The extreme tensile stress decreased less than other strain amplitudes and stabilized at a higher value. The compressive peak stress decreases slightly throughout the cycle and remains stable at a higher stress level. The hysteresis curve of the single-crystal iron at the 7% strain amplitude shows a more obvious cyclic hysteresis, as shown in Figure 2c. The peak tensile stress of monocrystalline iron at the 7% strain amplitude first softens and then stabilizes with the loaded strain, as shown in the red prisms in Figure 2d, which is similar to the stress level at the 6% strain amplitude. The compressive peak stress showed a slow decline trend in the 50 cycles, and the stress level was similar to the 6.5% strain amplitude at a higher value.

### 3.2. Polycrystalline Iron’s Mechanical Response Under Fatigue Conditions

**Figure 3:**
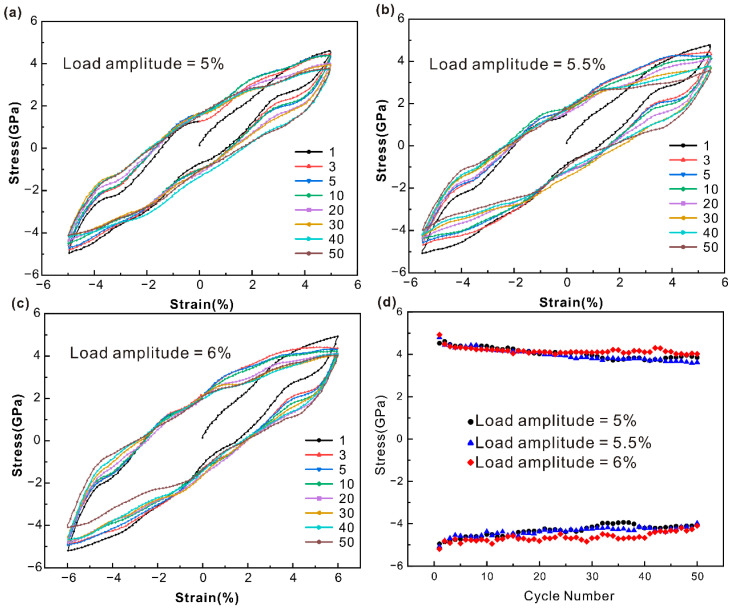
The cyclic stress–strain response curves of polycrystalline iron under diverse strain amplitude conditions of (**a**) 5%, (**b**) 5.5%, (**c**) 6%, and (**d**) the variation relationship of tensile and compressive peak stress with the number of cycles.

Figure 3 shows the cyclic stress–strain curves of a polycrystalline iron model at the 5%, 5.5%, and 6% strain amplitudes. The extensive area of the hysteresis loop suggests that the polycrystalline iron exhibits plastic deformation at a strain amplitude of 5%, as evident from Figure 3a. The peak tensile and compressive stresses of the 5% strain amplitude show a cyclic softening trend within 50 cycles, as shown by the black dots in Figure 3d. The hysteresis curve of the polycrystalline iron at the 5.5% strain amplitude appears to indicate cyclic hysteresis. Both tensile and compressive cyclic stress peaks show a trend of cyclic softening, as shown in the blue triangle in Figure 3d. The hysteresis curve of the polycrystalline iron at the 6% strain amplitude shows a more obvious cyclic hysteresis. The peak tensile stress is first cyclically softened and then stabilized at a higher value throughout the cycle, as shown by the red diamond in Figure 3d. The stabilized stress value of tensile peak stress is higher than that of the 5% and 5.5% strain. The peak compressive stress showed a tendency of cyclic softening during the whole period, and the compressive stress was also at a high value. This is consistent with the cyclic softening of iron-based alloys under tensile and compressive load offset as observed by Rosa et al. [40] through uniaxial sample experiments.

By comparing the hysteresis curves of the monocrystalline iron and polycrystalline iron, respectively, under three different strain amplitudes in Figure 2 and Figure 3, it can be seen that with the increase in strain amplitudes, plastic deformation begins to occur between the two and the phenomenon becomes more obvious with the increase in strain amplitudes. Both of them show the tendency of cyclic softening during the whole cyclic loading process. However, compared with the polycrystalline iron, the hysteresis of the monocrystalline iron not only occurs with the increase in strain amplitude during cyclic loading, but also distorts the hysteresis curve at the 30th cycle under the 6.5% strain amplitude and the fifth cycle under the 7% strain amplitude. Through the analysis of DXA, it can be seen that the reason for this phenomenon is that there are twins and detwinning occurs during the loading process, which distorts the hysteresis curve.

## 4. Discussion

### 4.1. Microstructure Evolution of Single-Crystal Iron Under Fatigue Load

#### 4.1.1. Plastic Deformation Behavior and Mechanism of Single-Crystal Iron

Dislocation slip [41,42,43], twinning [44] and phase transition [45,46,47] are the main plastic deformation mechanisms of monocrystal materials at room temperature. Based on the aforementioned three deformation mechanisms, this article will delve into the plastic deformation behavior of the single-crystal iron under varying strain amplitudes.

**Figure 4:**
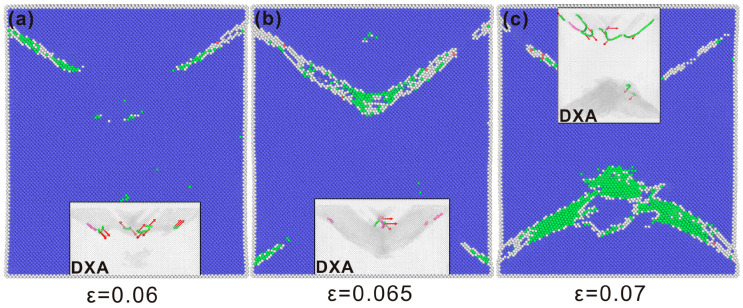
Tensile state of single-crystal iron at the first peak stress at three different strain amplitudes of (**a**) 6%, (**b**) 6.5%, (**c**) 7% (BCC atoms in blue and FCC atoms in green).

Figure 4 shows the state of the single-crystal iron when the tensile peak stress is reached for the first time at three distinct strain amplitude levels. Under the three strain amplitudes, the phase transition at the tensile peak begins at the edge of the crystal, and the dislocation also begins to initiate at the boundary and propagate into the crystal. It can be seen from the hysteresis curve (Figure 2) that there is basically no plastic deformation of the single-crystal iron under the strain amplitude of 6%. Therefore, the deformation behavior of the single-crystal iron under the 6.5% and 7% strain amplitudes is emphatically analyzed.

**Figure 5:**
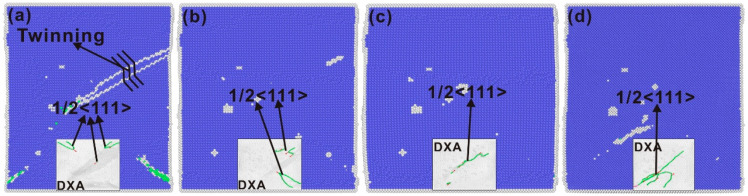
Phase transition and dislocation evolution of single-crystal iron at the 30th cycle at the 6.5% strain amplitude (**a**) 6.5% strain amplitude phenomenon during the 30th cycle of tensile, (**b**) 6.5% strain amplitude phenomenon during the first compression of the 30th cycle, (**c**) 6.5% strain amplitude phenomenon when the 30th cycle is recompressed, (**d**) 6.5% strain amplitude phenomenon when the 30th cycle compression is completed and the strain is stretched again (BCC atoms in blue and FCC atoms in green).

Figure 5 shows the phase transformation and dislocation evolution of the single-crystal iron at the 30th cycle at the 6.5% strain amplitude. As observed from the hysteresis curve in Figure 2b, the monocrystal iron has an abnormal compression after the 30th cyclic tensile extreme value, and the stress has an abnormal downward trend. Twin crystals appear along the inner slip plane $\left(1\bar{1}2\right)$ of the single-crystal iron at the 30th cyclic tensile extremum, as shown in Figure 5a. The internal stress is redistributed and the twin crystals disappear when the load is reversed. Owing to the reconfiguration of dislocations in the crystal during the detwinning phenomenon, the dislocations of the detwinning become entangled with each other to form long dislocation lines. As illustrated in Figure 5b–d, the plastic deformation of the single-crystal iron in a cycle is dominated by the 1/2<111> dislocation.

**Figure 6:**
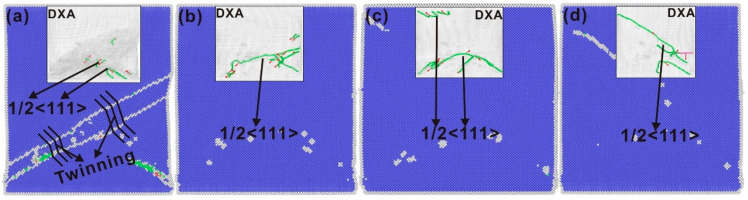
Phase transition and dislocation evolution of single-crystal iron in the third cycle at the 7% strain amplitude (**a**) 6.5% strain amplitude phenomenon during the 30th cycle of tensile, (**b**) 7% strain amplitude phenomenon during the first compression of the 30th cycle, (**c**) 7% strain amplitude phenomenon when the 30th cycle is recompressed, (**d**) 7% strain amplitude phenomenon when the 30th cycle compression is completed and the strain is stretched again (BCC atoms in blue and FCC atoms in green).

It can be seen from Figure 6a that with the increase in strain amplitude, twin crystals begin to appear along the slip plane (1–12) when the strain amplitude is 7% in the third cycle. In the subsequent cyclic loading process, constant twinning and detwinning phenomena lead to constant small fluctuations in the peak stress of tension and compression, which corresponds to the changing trend of the red diamond in Figure 2d. It can be seen from Figure 6b–d that at a strain amplitude of 7%, the dislocations caused by detwinned crystals begin to become entangled with each other, and the linearity of long dislocation lines becomes more obvious. The entire loading process is also dominated by the 1/2<111> dislocation. In summary, the edge dislocation within the region of localized plastic deformation plays the role of dislocation source under fatigue load and continuously emits dislocation crystals into the crystal. At the same time, with the increase in strain amplitude, twins appear and further promote the region of localized plastic deformation of the single-crystal iron. Irreversible plastic deformation gradually accumulates on the surface under cyclic load.

We observe that a phase transition from BCC-FCC occurs during the stretching process (Figure 4a–c). During the deformation process, FCC phase formation starts at the boundary and extends towards the center of the model. This is due to the fact that the crystal structure changes when the model is stretched, and some atoms rearrange to form the FCC structure. The metastable FCC structure does not appear during the transformation process of the single-crystal iron from a body-centered cubic (BCC) structure to a hexagonal close-packed (HCP) structure, according to previous studies [48]. Therefore, no BCC-FCC phase transition can be observed during the compression process.

#### 4.1.2. Evolution of Dislocation Number of Single-Crystal Iron Under Fatigue Load

The fatigue characteristics of metal materials are closely related to dislocation movement. During plastic deformation, moving dislocations can be propagated by Frank–Read sources to increase the dislocation density and thus, promote material plastic deformation. At the same time, when two opposing moving dislocations of Burgers vectors meet, they may annihilate each other, reducing the dislocation density. Therefore, it is necessary to analyze the dislocation of the single-crystal iron models loaded with different strain amplitudes.

**Figure 7:**
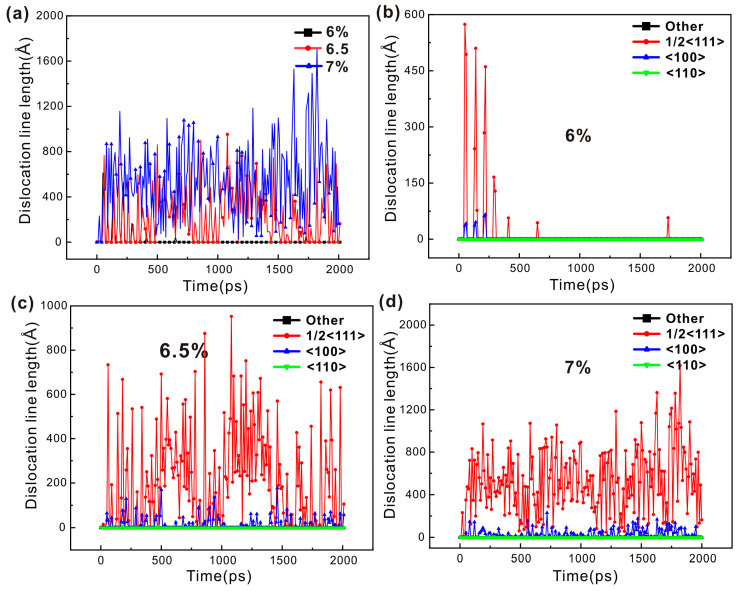
(**a**) The variation trend of the total dislocation line length in single-crystal iron across varying strain amplitudes; (**b**) variation trend of the length of four dislocation lines under the 6% strain amplitude; (**c**) variation trend of the length of four dislocation lines under the 6.5% strain amplitude; and (**d**) variation trend of the length of four dislocation lines under the 7% strain amplitude.

Dislocation density refers to the cumulative length of dislocation lines within a unit volume of crystalline material. Consequently, this study quantifies the variation in dislocation density of the single-crystal iron under loading conditions by assessing the length of dislocation lines. As illustrated in Figure 7a, as the strain amplitude increases, the total length of the dislocation lines progressively rises, which is a finding that aligns with the conclusions reached by other researchers. Figure 7a shows the trend in total dislocation line length for the single-crystal iron at three distinct strain amplitudes. Within a single cycle, the length of the dislocation line varies due to the partial annihilation of dislocations generated during forward loading when subjected to backward loading. The level of the dislocation line length is the remaining dislocation and stratification in the crystal, and the annihilation of the dislocation indicates the appearance of irreversible deformation. The single-crystal iron has only a few dislocations at the 6% strain amplitude. The total dislocation line length of the single-crystal iron decreases at first and then increases at the 6.5% strain amplitude, then decreases again and fluctuates steadily. The total dislocation line length of the single-crystal iron increases at the 7% strain amplitude and fluctuates at a higher level after reaching a certain value. It can be seen from the hysteresis curve and the total dislocation line length of the single-crystal iron that only a small degree of plastic deformation occurs under the strain amplitude of 6%. The single-crystal iron has a significant extent of plastic deformation under the strain amplitudes of 6.5% and 7%.

Figure 7b–d show the variation in the length of three categories of dislocations of the single-crystal iron with fatigue loading under three strain amplitudes. From this, it is possible to conduct a qualitative analysis of the relationship between dislocation features and the cyclic softening and saturation behaviors observed in the mechanical properties of the single-crystal iron. The initial phase of fatigue dislocation 1/2<111> of the single-crystal iron at the 6.5% strain amplitude increases rapidly, and the plastic deformation behavior in the crystal is dominated by it. The <100> dislocation and <110> dislocation begin to appear as the loading continues, but the number of both is very small, so the subsequent plastic deformation process is still dominated by the 1/2<111> dislocation. Due to the phenomenon of twinning and detwinning, the number of dislocations at this time decreased significantly during loading in the 30th to the 35th cycle and then remained stable. The number of 1/2<111> dislocations of the single-crystal iron at the 7% strain is significantly higher than that at the 6.5% strain, which promotes the plastic deformation of the crystal. At the same time, it also reflects that the dislocation of the single-crystal iron increases continuously and the entanglement becomes more intense during plastic deformation, and the reversibility of the dislocation motion decreases. The tensile extreme stress is lower than the 6.5% load condition due to the larger density of the dislocation during the tensile extreme. At the same time, the twin formed by the single-crystal iron under the compression load causes the dislocations to tangle with each other, which makes the extreme compressive stress stable at a high level. The twin boundary becomes more and more obvious in restricting the slip of the movable dislocation as the loading continues, resulting in the diastole slip of the movable dislocation along the twin boundary, resulting in constant dislocation pinning and accumulation at the twin boundary. Therefore, the number of dislocations only increases at the beginning of loading and then maintains a high level with small fluctuations. In general, the single-crystal iron has plastic deformation under both the 6.5% and 7% strain amplitudes and is dominated by the 1/2<111> dislocation. But the latter has more dislocation, greater density and more obvious plastic deformation due to the presence of twins. Chen et al. [49] studied the low-cycle fatigue behavior of TWIP steel under cyclic load and used the EBSD method to analyze the microstructure characteristics of the sample after cyclic load; they found that a large number of continuous dislocation movements in the initial stage of the plastic deformation of the material led to the plastic deformation of the material, and twin crystals also appeared during the deformation process. Dislocation dominates the entire deformation process, which corresponds to the results of the single-crystal iron.

### 4.2. The Microstructural Evolution of Polycrystalline Iron Subjected to Fatigue Loading

#### 4.2.1. Plastic Deformation Behavior of Polycrystalline Iron Subjected to Fatigue Loading

**Figure 8:**
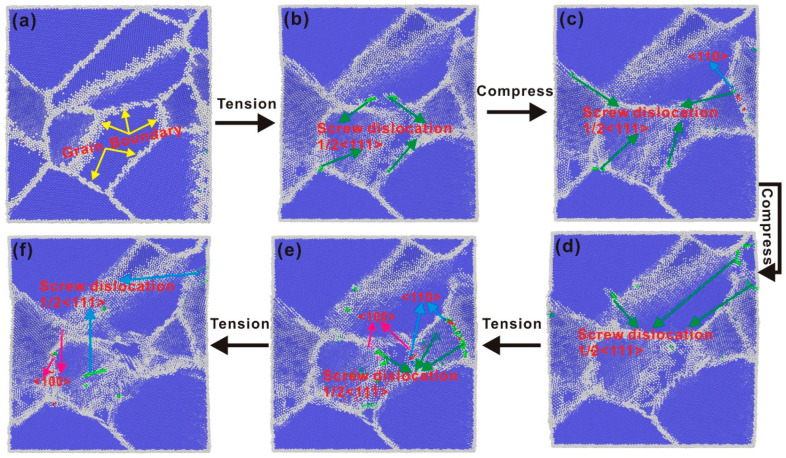
Changes in grains and dislocations of polycrystalline iron in one period at the 6% strain (**a**) The unchanged initial state of polycrystalline iron, (**b**) Polycrystalline iron begins to stretch for the first time, (**c**) Polycrystalline iron begins to compress for the first time, (**d**) The polycrystalline iron begins to compress a second time, (**e**) Polycrystalline iron begins to stretch a second time, (**f**) Polycrystalline iron completes a cycle(the yellow arrow is the grain boundary, the green arrow is the 1/2<111> dislocation, the blue arrow is the <110> dislocation, and the pink arrow is the <100> dislocation).

Figure 8 shows the dislocation evolution of the polycrystalline iron through a complete cycle at a strain amplitude of 7%. At the initial stage, it is observed that no dislocation occurs in the polycrystalline iron. A perfect screw dislocation of 1/2<111> at the beginning of the stretch nucleates from the grain boundary, as shown by the green arrow in Figure 8b. This shows that the perfect spiral dislocation is prone to slip inside the grain, which also results in a decrease in the resistance inside the polycrystalline iron, resulting in the beginning of a decrease in stress. Other defects such as the <100> (pink arrow in Figure 8e) dislocations at the boundary and <110> (blue arrow in Figure 8b) begin to appear as the tensile strain increases when the polycrystalline iron begins to deform plastically. The maximum deformation occurs at the grain boundary, and no large deformation occurs within the grain during the initial deformation process, as can be seen from Figure 8b. This indicates that the grain boundary is the main source of defects. The additional strain in the compression direction results in the development of a new 1/2<111> perfect screw dislocation (Figure 8d) when the polycrystalline iron begins to be compressed. The dislocation moves and extends to the other side of the grain boundary (Figure 8e) as the compressive strain increases. No twin formation was observed during the strain in the compression direction (Figure 8c,d). Apart from the initial loading and unloading processes, no FCC phase formation was noted during the subsequent cyclic deformation. Various dislocations occurring within the grain will revert to the grain boundary regions (Figure 8e) when the compression load is unloaded and the tensile load reappears. In addition, the direction of the perfect screw dislocation of 1/2<111> during redrawing is similar, as shown in Figure 8e,f. However, the direction of the dislocation is reversed during loading, that is, the 1/2<111> perfect screw dislocation moves from the grain boundary in the opposite direction. Upon additional stretching, the 1/2<111> perfect screw dislocations initiate at various positions and spread along their respective paths, following the regions of maximum stress.

#### 4.2.2. Evolution of Dislocation Number of Polycrystalline Iron Under Fatigue Load

**Figure 9:**
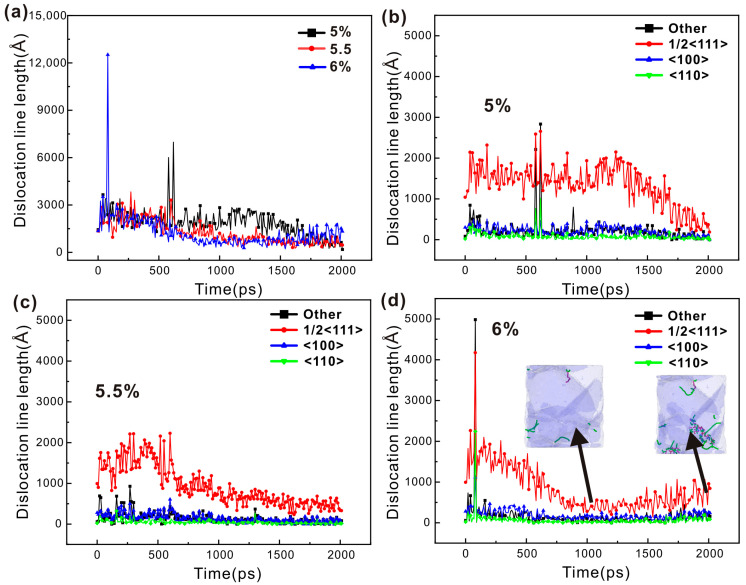
(**a**) The changes in the total length of dislocation lines within polycrystalline iron across three unique strain amplitude levels; (**b**) the changes in the length of four dislocation lines at a strain amplitude of 5%; (**c**) the changes in the length of four dislocation lines at a strain amplitude of 5.5%; and (**d**) the changes in the length of four dislocation lines at a strain amplitude of 6%.

The dislocation density analysis of the polycrystalline iron was also performed as was done with monocrystalline iron. Compared with Figure 7 and Figure 9, it can be seen that the total dislocation line length of the polycrystalline iron is significantly longer and the dislocation density is greater than that of the single-crystal iron. This shows that the dislocation movement and plastic deformation behavior of the polycrystalline iron are more complicated than that of the single-crystalline iron during loading. Figure 9a shows the variation trend of the cumulative length of the dislocation lines of the polycrystalline iron under three strain amplitudes. It can be seen from the figure that the dislocation line length of the polycrystalline iron does not show the phenomenon like that of the single-crystalline iron, where the total dislocation line length increases successively as the strain amplitude increases. This may be due to the accumulation of dislocations at the grain boundaries because grain boundaries prevent the long-distance movement of dislocations. And due to the influence of grain size, the polycrystalline iron is more inclined to achieve plastic deformation through grain boundary migration and grain rotation than through dislocation slip [50]. These two possibilities are discussed below. The total dislocation line length of the polycrystalline iron at the 5% strain amplitude initially increased and then began to decrease, due to the sudden increase in <110> dislocation, the peak value increased, and the stable fluctuation finally continued to decrease. The total dislocation line length of the polycrystalline iron increases initially at the 5.5% strain amplitude and then decreases until it fluctuates smoothly. The total dislocation line of the 6% strain amplitude initially increases, then decreases, and then increases again. It can be seen, according to the hysteresis curve and the total dislocation length of the polycrystalline iron, that the polycrystalline iron has obvious plastic deformation and cyclic softening properties under the three strain amplitudes.

In order to qualitatively determine the relationship between the characteristics of polycrystalline iron dislocations and cyclic softening and saturation, the variation in the length of each type of dislocation with fatigue load is shown in Figure 9b–d. The length of the 1/2<111> dislocation line stabilizes at a high value in the initial stage under the three strain amplitudes. This is a dislocation at the grain boundary. The 1/2<111> dislocation dominates the plastic deformation of the crystal, and the polycrystalline iron begins to undergo cyclic softening. The dislocation line length in 1/2<111> crystal direction is still high at the 5.5% strain amplitude, but the gap with other crystal directions is reduced, which indicates that the slip system of other crystal directions also begins to participate in the dislocation activity. The phenomenon, similar to the 5.5% strain amplitude, also appears at the 6% strain amplitude. This indicates that with the increase in strain amplitude, the slip systems of other crystal directions also begin to participate in the dislocation activity, namely, the <100> and <110> dislocations. However, by comparing the change of the 1/2<111> dislocation line length under the three strain amplitudes, it can be seen that with the increase in strain amplitude, the grain boundary merging speed of the polycrystalline iron becomes faster, resulting in the faster disappearance of the accumulated dislocation at the grain boundary, and the time point of dislocation reduction is advanced. In general, the polycrystalline iron has obvious plastic deformation under the three strain amplitudes, and the number of 1/2<111> dislocations in the deformation process is the largest. However, different from the single-crystal iron, other crystal orientation dislocations, such as <110> and <100>, begin to participate in the deformation process. And the deformation of the polycrystalline iron becomes more complicated due to the grain boundary obstruction and the influence of grain size. Teramae et al. [51] observed the low-cycle fatigue behavior of SN490B steel under cyclic load through EBSD and observed that there was significant cyclic softening and multiple slip systems were in active states.

#### 4.2.3. Change in Grain Size of Polycrystalline Iron Under Fatigue Load

**Figure 10:**
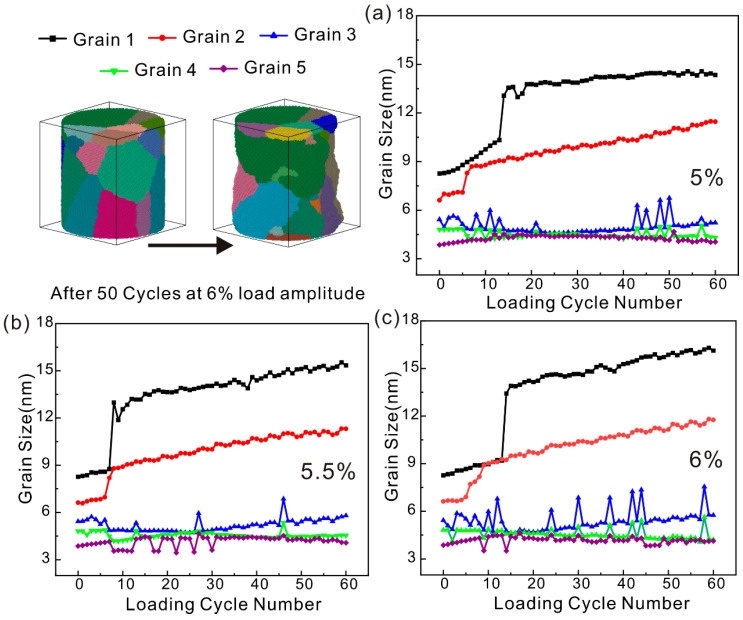
Growth of grain size of polycrystalline iron under fatigue load. (**a**) 5% cyclic load, (**b**) 5.5% cyclic load, and (**c**) 6% cyclic load.

As depicted in Figure 10, schematic illustrations of the grain size of polycrystalline iron at the initiation and termination of cyclic loading under a 6% strain amplitude are, respectively, presented. After the end of the cycle load, the grains in the polycrystalline iron grow significantly. Polycrystalline iron consists of grains with varying orientations, and the grain boundaries between these differently oriented grains facilitate the fusion of individual grains. At the grain boundaries, the presence of grains with varying orientations leads to the formation of a significant number of dislocations and disordered atoms. Under cyclic loading, there is less internal dislocation emission by the polycrystalline iron, and the plastic deformation is dominated by grain boundary movement. The grains rotate under cyclic loading, and adjacent grains tend to merge. The progressive coalescence of multiple grains is inclined to form two larger grains, as shown in Figure 10a–c by the black squares and red dots. The grain size given in this paper is regarded as a cube, and the side length of the cube is given according to the grain volume calculated by OVITO. The original grain size of the polycrystalline iron can be as large as 8 nm, and the maximum grain size gradually increases at the 5% strain amplitude and finally reaches about 15.1 nm. The maximum grain size is 15.6 nm at the 5.5% strain amplitude, and some small grains merge to form two larger grains. The plastic deformation at the 6% strain amplitude is dominated by two grains. After 20 cycles, the dominant grain size increases abruptly, and the final grain size reaches 16.5 nm.

Comparing the grain size changes under the three strain amplitudes, it can be seen that the grain fusion degree is smaller under the 5% strain amplitude. The fusion of grains will lead to the disappearance of mismatch dislocations at the grain boundary, and the number of dislocations will gradually decrease. The grain size changes obviously at the 5.5% and 6% strain amplitudes. According to the dislocation changes, it can be seen that the initial dislocation number of the two is basically the same and begins to decrease, which is because the grains in the crystal begin to fuse. Due to the small strain amplitude at 5.5%, the number of dislocations in the cyclic loading process is dominated by grain fusion and eventually remains stable. However, the number of dislocations at the 6% strain amplitude decreased and then began to increase. This shows that the plastic deformation changes from grain movement to the proliferation of dislocations in the grain. Although the grain size of the two has no obvious change after fusion, the deformation mechanism has changed fundamentally.

#### 4.2.4. The Buildup of Localized Plastic Deformation in Polycrystalline Iron Under Cyclic Loading

**Figure 11:**
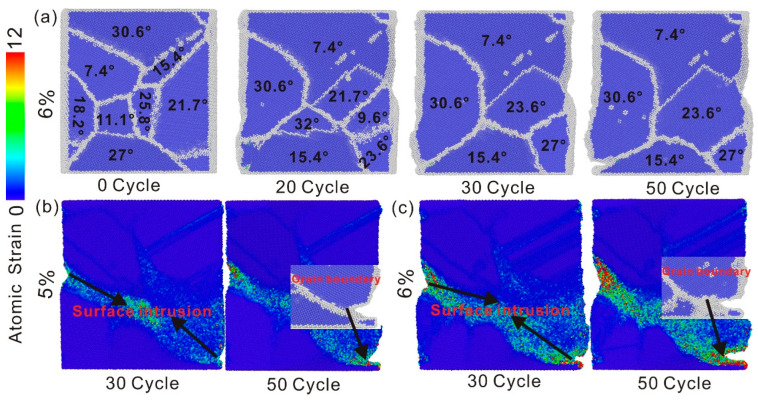
(**a**) Grain orientation change at the 6% strain amplitude, (**b**) atomic stress characterization and surface intrusion of the YOZ section at the 5% strain amplitude, and (**c**) atomic stress characterization and surface intrusion of the YOZ section at the 6% strain amplitude.

In general, plastic deformation is carried out by grain boundary sliding, dislocation movement, or entanglement. But either mechanism can lead to plastic deformation. It can be observed from the variations in the angles of distinct grains that grain boundary sliding and grain boundary rotation take place during the deformation process of polycrystalline iron, as depicted in Figure 11a. The adjacent grains merge with each other during the movement to complete the grain growth. The rotation of the grain no longer occurs when the grain grows to a larger size, and the merger process is accomplished through the rotational movement of the surrounding smaller grains. Figure 11b,c show the stress concentration and surface intrusion of the YOZ section at the 5% and 6% strain amplitudes. The degree of stress concentration increases with the increase in strain amplitude, and all of them appear at the grain boundary. The increase in grain size indirectly reflects the degree of grain boundary movement. The rotation of grains causes the local plastic accumulation to occur at the grain boundary due to the different orientation of adjacent grains. If the adjacent grains do not complete fusion through rotation when the grain size increases, the plasticity at the grain boundaries between them will continuously accumulate. The cyclic load tends to form local stress concentration at the grain boundary, and the increase in external load will aggravate the stress concentration and cause surface intrusion. The accumulation process is intensified and surface intrusion occurs at the stress concentration at the grain boundary at the 5% and 6% strain amplitudes. Plastic deformation gradually accumulates and fatigue damage is caused at the grain boundary with the action of cyclic load. This means that the grain boundary is the preferred location for the fatigue damage of polycrystalline iron.

## 5. Conclusions

In this paper, by analyzing the fatigue mechanical properties and microevolution mechanisms of single-/polycrystalline iron under three different strain amplitudes, the following conclusions are obtained.

1. The irreversible dislocation and layer fault of single-crystal iron under cyclic load are the initial structure of local plastic deformation, and they play the role of dislocation source. The accumulation of local plasticity occurs in the local region where the slip planes cross. The 1/2<111> dislocation plays an important role in the plastic deformation of single-crystal iron.
2. Twin crystals formed by single-crystal iron under fatigue load are microstructures that are resistant to local plastic accumulation.
3. The grains of polycrystalline iron rotate and merge with each other to complete the grain growth under cyclic loading. Due to the different orientation of adjacent grains, the accumulation of local plasticity is mainly located at the grain boundary due to the rotation of each grain. The consolidation of grains is accomplished by the rotation of nearby smaller grains when a single grain is large enough. If the surrounding grains cannot be merged through rotation, plastic accumulation at the grain boundary results in fatigue damage.
4. Polycrystalline iron forms stress concentration and surface intrusion at grain boundaries under cyclic loading, and the distribution of atomic strain increases with the increase in the number of cycles. The greater the strain amplitude, the more serious the local plastic accumulation. The competition of these mechanisms causes the mechanical properties of polycrystalline iron to soften under cyclic loading.

## Data Availability

The data for this article, including [Potential function] are available at https://www.ctcms.nist.gov/potentials/system/Fe/#Fe (accessed on 10 January 2022).
